# Laparoscopic Transabdominal Preperitoneal Repair for a Recurrent Inguinal Hernia After Direct Kugel Procedure: A Case Report

**DOI:** 10.7759/cureus.117108

**Published:** 2026-09-28

**Authors:** Hirokazu Matsuura, Kotaro Nishida, Taro Kataoka, Takayuki Okuno, Yusuke Kyoden

**Affiliations:** 1 Department of Surgery, Ibaraki Prefectural Central Hospital, Ibaraki, JPN; 2 Department of Surgery, Kitaibaraki City Hospital, Ibaraki, JPN

**Keywords:** case report, direct kugel, laparoscopic surgery, laparoscopic transabdominal preperitoneal repair, recurrent inguinal hernia

## Abstract

Recurrent inguinal hernia (IH) repair is difficult and challenging because of the anatomical complexity and alterations involved. Recurrence is a multifactorial outcome influenced by both surgical and anatomical factors. Adopting a meticulous approach is important to avoid complications and recurrence. There is no consensus regarding the optimal approach for repairing recurrence. A laparoscopic approach is often preferred, as it provides clear visualization and facilitates identification. This report describes the case of a 78-year-old male patient, treated with direct Kugel (DK) hernioplasty three years earlier, who underwent laparoscopic surgery for recurrence. Completely covering the myopectineal orifice (MPO) with adequate overlap was essential to preventing recurrence. Laparoscopic surgery offers a beneficial approach for such cases.

## Introduction

Inguinal hernia (IH) is a common disease, and its treatment is surgical intervention [1]. The number of laparoscopic hernioplasty cases in Japan has been increasing. Although advances in surgical techniques and instruments have been introduced to increase success rates and reduce complications, the incidence of recurrence remains unchanged. A different approach is recommended for a recurrent IH, depending on which technique was used in the primary surgery. Because the laparoscopic approach can be performed in a new layer, it is recommended after a failed anterior approach. The direct Kugel (DK) technique is an anterior approach that accesses the preperitoneal space without laparoscopic assistance, and the postoperative quality of life of these patients is usually high [2]. However, there have been several reports of recurrence.

Performing laparoscopic repair for recurrence following DK procedures is highly challenging because of extensive adhesions in the preperitoneal space. The preperitoneal space is compromised by previous surgical interventions and the presence of mesh [3]. When a laparoscopic approach is selected, there are certain advantages, particularly when an extensive preperitoneal space is required, which is occasionally closed using the medial umbilical ligament [4]. We present a case of transabdominal preperitoneal (TAPP) repair in a patient with a recurrent IH following repair with the DK procedure.

## Case presentation

A 78-year-old male presented to our hospital with a recurrent left IH. The patient had undergone DK hernioplasty three years prior for a bilateral IH. Physical examination revealed a fist-sized bulge in the left groin area, along with 5 cm incisional wounds in both inguinal regions. CT scans revealed a recurrent left IH with small intestine as the hernia content (Figure 1). Given the possibility of difficulty with peritoneal closure, we opted for TAPP using an anti-adhesion-coated mesh.

**Figure 1:**
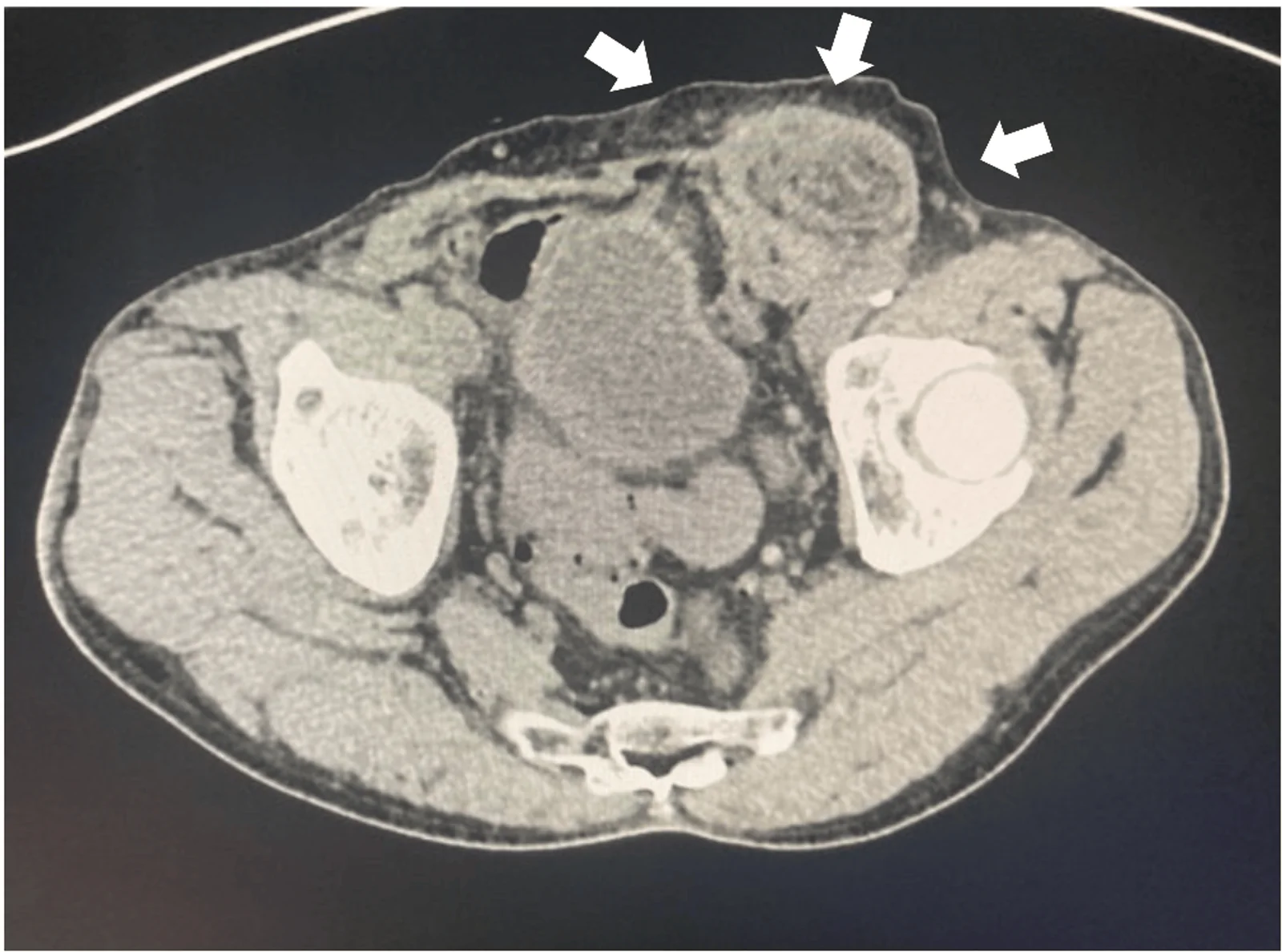
CT scan showing a left inguinal hernia (white arrows) CT: computed tomography

Under general anesthesia, the patient was positioned supine, with his head down and left side up. A 12-mm transumbilical port for the camera and two 5-mm working ports were inserted on the right and left sides of the abdomen. Laparoscopic observation led to a diagnosis of RL3 according to the European Hernia Society classification. The mesh from the initial surgery was located at the posterior and medial side of the hernia orifice. We assumed that the recurrence had occurred as a result of corrugation and displacement of the initial mesh. The peritoneum was opened circularly from the anterior superior iliac spine to the medial umbilical ligament. Subsequently, meticulous dissection around the testicular vessels and vas deferens was performed to expose the internal inguinal ring and the myopectineal orifice (MPO). The area where the mesh had been placed showed scarring and severe hardening, making detachment difficult. We did not remove the previously placed mesh, and the peritoneum was dissected to fully expose the MPO (Figure 2).

**Figure 2:**
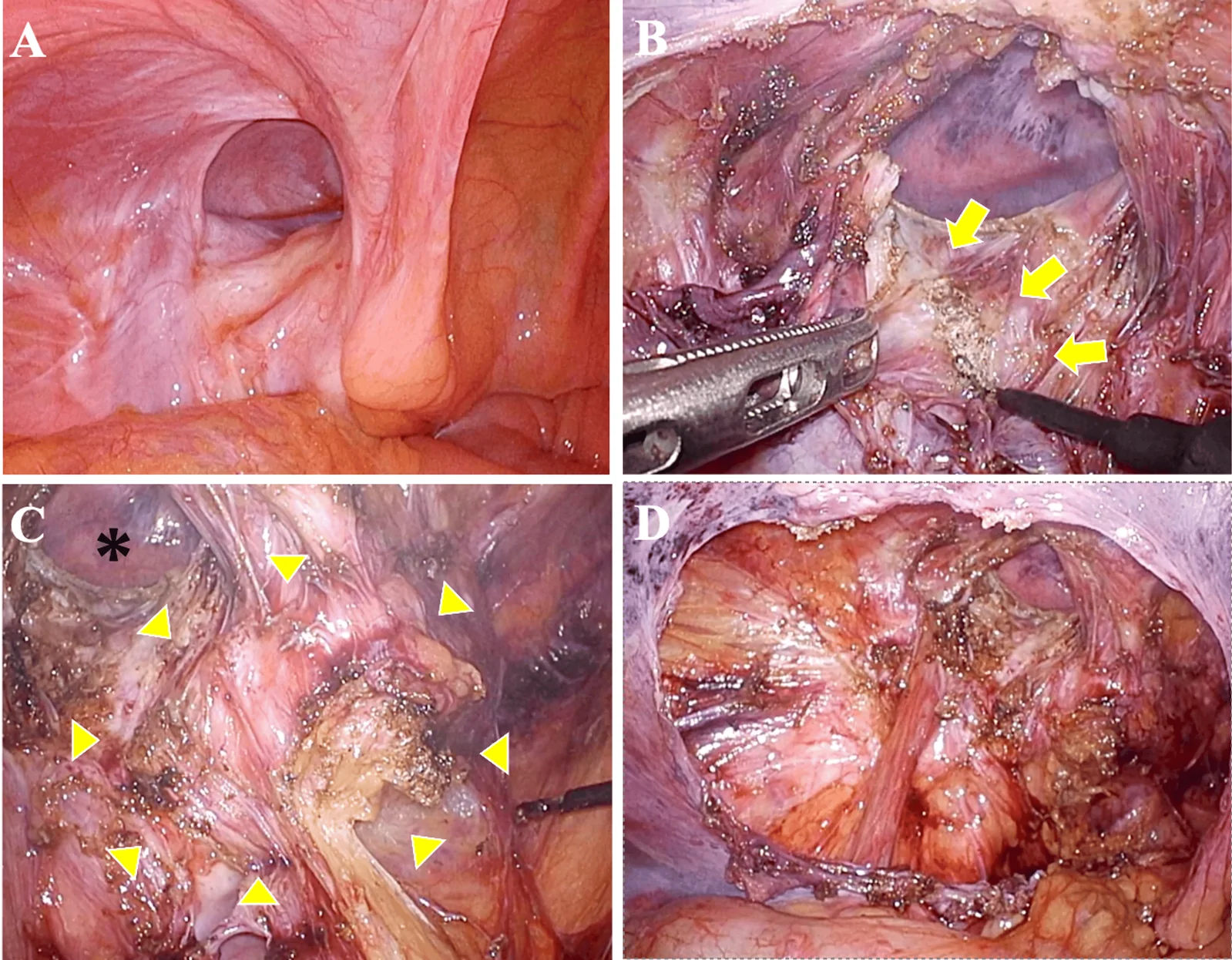
TAPP repair for recurrence (A) Laparoscopic observation; (B) severe scarring between the mesh (yellow arrow) and the peritoneum; (C) the mesh from the initial surgery (yellow arrowhead) located on the posterior and medial side of the hernia orifice (＊); and (D) the MPO after meticulous dissection TAPP: transabdominal preperitoneal; MPO: myopectineal orifice

Following the preparation of the mesh placement area, the mesh (Bard™ Composix™ L/P Mesh 10.8 cm × 15.9 cm; Davol Inc., Tiverton, RI) with trimming was inserted into the abdomen and spread to cover the MPO. Then, the mesh was fixed to the Cooper ligament, rectus muscle, and both sides of the inferior epigastric vessels using absorbable tackers. Trimming made it possible to avoid danger zones and important neurovascular structures. We sutured the mesh in the area of overlap (Figure 3). Given the prolapsed peritoneum and the peritoneal defect, in conjunction with severe adhesion, the primary peritoneal closure was impossible. Peritoneal closure was executed by using the medial umbilical fold. The peritoneum was sutured without any defects, and the mesh was covered (Figure 4). Subsequently, the port was removed, and the abdominal incision was closed. 

**Figure 3:**
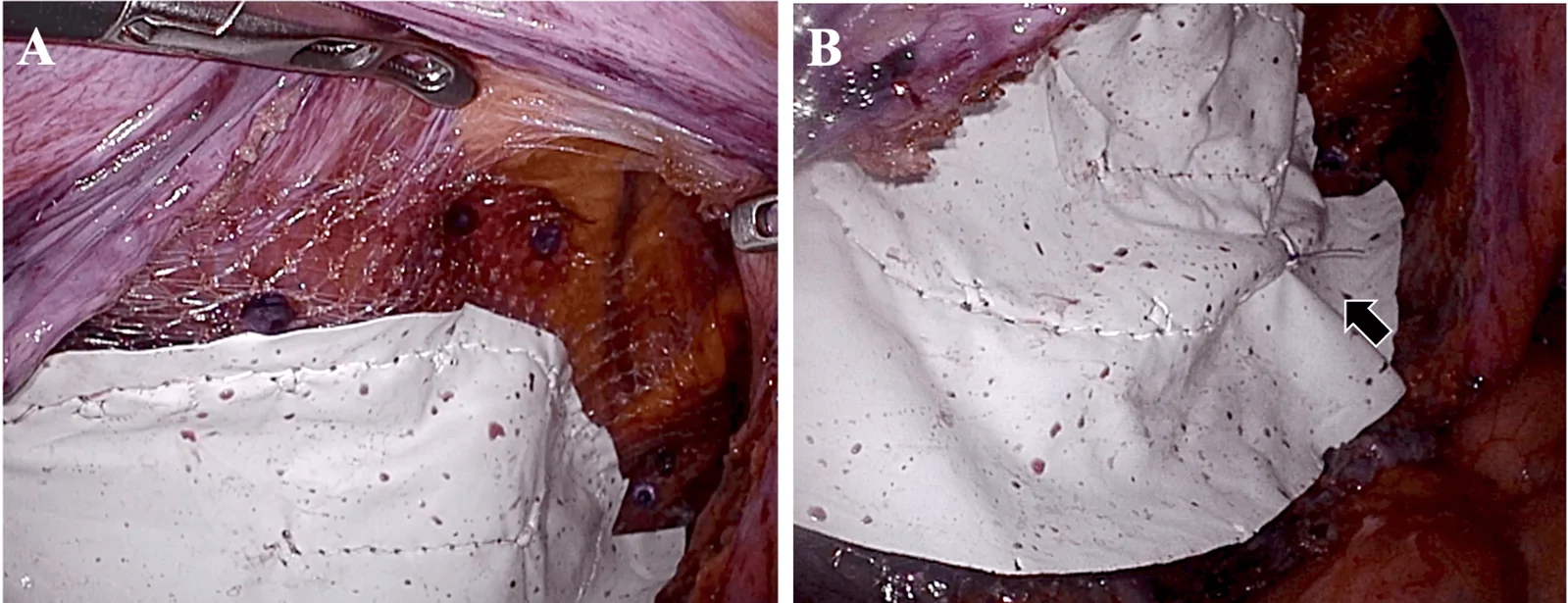
The anti-adhesion-coated mesh covering the MPO The mesh (A) was fixed under visual control after trimming, and (B) was sutured to each other in the area of overlap (black arrow) MPO: myopectineal orifice

**Figure 4:**
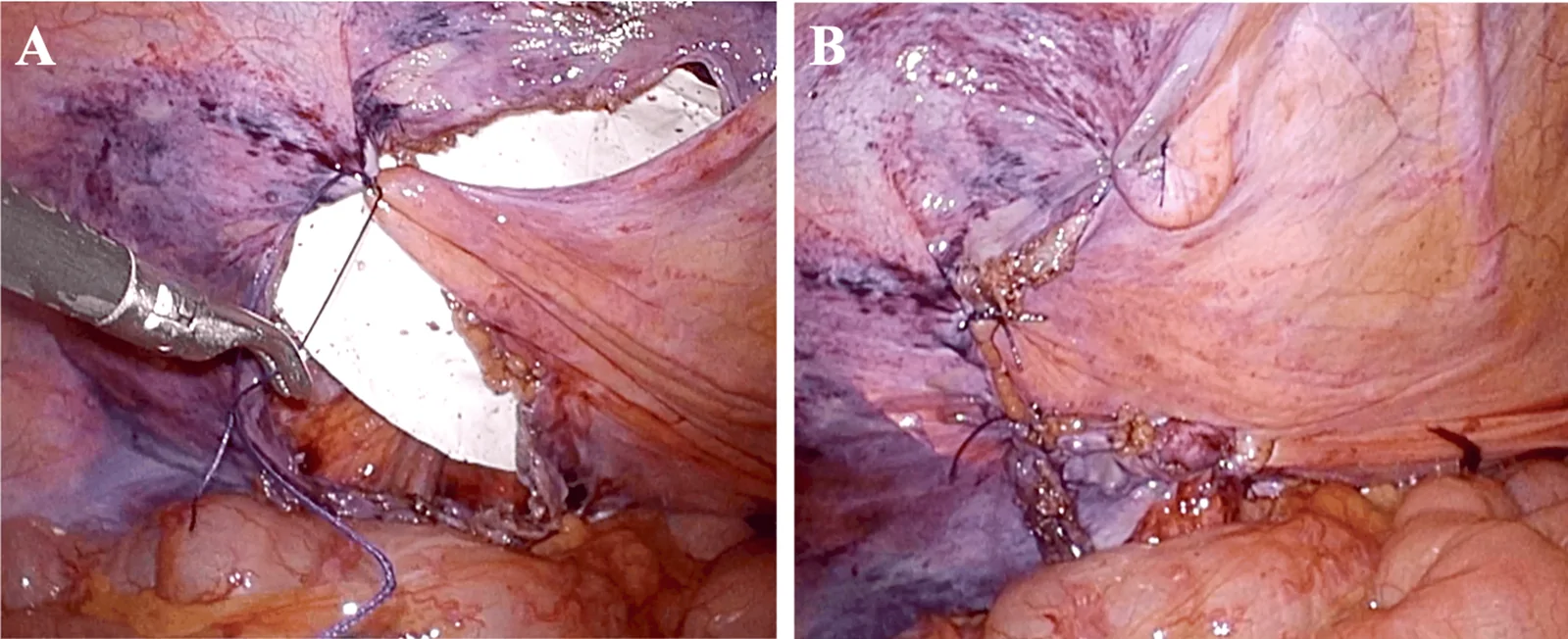
Peritoneal closure (A) Using the medial umbilical fold and (B) covering without any defects

The total surgical time was 148 minutes with minimal blood loss. The patient had an uneventful recovery and was discharged on the first postoperative day. No recurrence occurred during one year of follow-up.

## Discussion

The blind mesh placement in the DK procedure may have contributed to the recurrence. Although the exact recurrence rate after using the Kugel technique is uncertain, it has been reported to be 0-7.7% [2]. Recurrence can be attributed to inadequate coverage of the hernia ring or insufficient distribution of the mesh. In this case, the mesh was corrugated and shifted toward the medial side, possibly resulting in indirect inguinal recurrence. We express a preference for TAPP in cases of recurrent hernias.

Meta-analysis studies have shown that laparoscopic repair is associated with significantly less pain, a shorter duration of convalescence, and significantly fewer hematomas and seromas. The incidence of nerve and vessel injury was lower in the TAPP group than in the anterior surgery group [3]. The magnified laparoscopic view allows identification of anatomical landmarks, such as the spermatic duct and testicular vessels. The greatest challenges in recurrent hernia repair are adhesions and abnormal anatomy resulting from previous surgery [4]. This precise identification contributed to a successful TAPP repair.

Furthermore, laparoscopy makes it possible to confirm the adequacy of the newly inserted mesh [5]. This information cannot be obtained through anterior observation alone. Laparoscopic surgery requires exposure of the MPO and adequate mesh coverage. In fact, sufficiently covering the MPO with an overlap of more than 2 cm has been shown to achieve a drastic reduction in the recurrence rate, from 6.2% to 0.7% [6]. Kapiris et al. reported that a 5% recurrence rate with an 11 × 6 cm mesh decreased to 0.16% with a 15 × 10 cm mesh [7]. Mesh size sufficient to fully cover the MPO is effective in preventing recurrence. While both approaches are challenging following a DK procedure, TAPP was selected for these reasons.

TAPP is also advantageous for changing approaches, including intraperitoneal onlay mesh repair or an anterior approach, if needed. This hybrid approach allows precise identification and dissection of the hernia sac [5]. If laparoscopic hernia repair is difficult because of tissue scarring, a hybrid approach may be effective. Nitta et al. reported that robotic TAPP is effective for recurrence even when the space has severe adhesions [8]. The advantages of robotic technology, such as detailed visualization in three dimensions and an increased range of instrument motion, may expand the application of minimally invasive hernia repair to complicated cases.

Removing previously placed mesh should be avoided, as it increases the risk of injury to normal structures and results in substantial peritoneal defects. In this case, it was not possible to separate the peritoneum from the previous mesh because of the severity of the scarring around it. Because our focus was on coverage, the MPO could be covered with a new mesh placed over the previous mesh. The meshes were fixed securely with sutures. To achieve total peritoneal closure, use of an anti-adhesion-coated mesh or the medial umbilical fold is necessary.

We selected the anti-adhesion-coated mesh because complete peritoneal coverage was uncertain to achieve. An anti-adhesion-coated mesh is preferable to a normal mesh when peritoneal closure is difficult. Following trimming of the mesh, fixation using tackers was successful under direct visualization. Our technique is most effective when an anti-adhesion-coated mesh is used.

For recurrent hernias, the optimal procedure should be selected based on each case, taking into account the details of the previous surgery, the patient's general condition, the form of the recurrence, and the practitioner's skill level. We selected the TAPP technique to address this recurrence.

## Conclusions

This report describes a case of TAPP in a patient with a recurrent IH following a DK procedure. Laparoscopy enables confirmation of MPO exposure and adequate mesh coverage when performed by surgeons experienced in redo preperitoneal anatomy. Our techniques, which include covering the MPO entirely with adequate overlap, using an anti-adhesion-coated mesh, and achieving total peritoneal closure with the medial umbilical fold, are feasible options for preventing recurrence. The use of an anti-adhesion-coated mesh required trimming and suturing at the area of overlap. TAPP offered improved visualization, reduced tissue trauma, and enhanced precision. In general, the laparoscopic approach is considered reasonable for managing recurrence.
